# The Relationship Between Searching for Information on Dairy Products, Their Consumption, and Attitudes Towards New Dairy Products

**DOI:** 10.3390/foods15040695

**Published:** 2026-02-13

**Authors:** Marzena Jeżewska-Zychowicz, Marta Sajdakowska

**Affiliations:** Department of Food Market and Consumer Research, Institute of Human Nutrition Sciences, Warsaw University of Life Sciences (SGGW-WULS), 159C Nowoursynowska Street, 02-787 Warsaw, Poland

**Keywords:** dairy consumption, attitude, food neophobia, sources of information, dairy market

## Abstract

The increase in the market offer of dairy products may encourage their greater consumption. The study aimed to assess the relationship between dairy product consumption, searching for information about dairy products, and attitudes toward new dairy products. It was conducted in December 2024 and January 2025 using Computer-Assisted Web Interviewing (CAWI) among a representative sample of 1006 adult Poles. Based on the daily dairy consumption frequency, individuals who rarely consumed dairy products (below the median) and those who frequently consumed dairy products (at or above the median) were identified. Using exploratory factor analysis (EFA), two factors describing attitudes toward new dairy products were identified: “Openness to new dairy products” and “Concerns about new dairy products”. Based on these factors, cluster analysis identified three homogenous groups: “Moderate innovativeness and low concerns”, “Low innovativeness and medium concerns”, and “High innovativeness and high concerns”. The Chi-square test and Kruskal–Wallis test were used to evaluate the relationships between variables at *p* < 0.05. Among the “High innovativeness and high concerns” and “Moderate innovativeness and low concerns” clusters, there were fewer people who rarely consumed dairy products than those who frequently consumed them (38.1% vs. 61.9% and 45.4% vs. 54.6%, respectively). “High innovativeness and high concerns” consulted various information sources more often. Greater openness to new dairy products was associated with higher consumption of dairy products, regardless of the level of concern about these products. Openness to new dairy products, along with great concerns about them, was associated with more frequent use of specific information sources about dairy products. The research results confirm the relationship between dairy product consumption and openness to new dairy products, as well as the relationship between the latter and the search for information about dairy products, which may be of significant importance for future improvement of the situation on the dairy market.

## 1. Introduction

Consuming dairy products may help prevent various diseases, including osteoporosis [1,2], cardiovascular issues, and some cancers [3]. Some studies have also linked dairy consumption with improved body composition, lower rates of type 2 diabetes, and stronger muscles [4,5]. Research has also shown that people are aware of the benefits of consuming dairy products, but a significant portion of the world’s population still does not consume the recommended amount of these products [6,7,8]. Moreover, the consumption of dairy products varies due to socio-demographic characteristics such as age [6], gender [6,9,10,11], education [6], place of residence [11], and household size [12]. The demonstrated differences in dairy product consumption across population groups indicate that not only should compliance with recommendations regarding dairy product consumption be improved, but also that socio-demographic factors should be taken into account to enhance the effectiveness of messages aimed at increasing the volume and structure of dairy product consumption.

In addition to consuming dairy products at levels below the recommended amount, a decrease in their consumption is also observed [7,13]. The main reasons for limiting dairy consumption are health-related concerns, i.e., the desire to reduce fat, cholesterol, and lactose intake [14,15]. Reported difficulty digesting certain dairy products may also be a significant reason for limiting dairy consumption, especially cheese [7]. Moreover, ecological and animal welfare arguments are increasingly highlighted as reasons to avoid milk [13,16]. As in many other countries [13,17], a decline in milk consumption has also been observed in Poland over the past two decades, from 64.7 L per person per year in 2000 to 34.2 L per person per year in 2022. In contrast, the consumption of dairy products, such as yogurt, cheese, cottage cheese, and sour and sweet cream, has remained constant in Poland (approximately 22 L per person per year) [18]. In other countries, however, parallel to a decline in milk consumption, there is a growing consumption of different dairy products (i.e., cheese, yogurt) [19]. Additionally, consumption of new categories of sweetened beverages, including drinking yogurts and flavored milk, is increasing as well [20].

The dairy industry is currently undergoing significant changes due to technological advancements, growing consumer health awareness, and environmental concerns, among other factors [21]. Dynamic market changes and high competitiveness mean that the success of new food products is crucial for the well-being of producers [22]. However, the food industry is characterized by a high rate of new product failure [22,23]. This may be a result of consumers being more cautious about what they eat. Unlike other products, food enters the body and affects the health. Thus, the fear of new foods, which is the essence of food neophobia [24], can hinder the acceptance of new products. Although this phenomenon is widespread in children, food neophobia can persist into adulthood and influence adults’ decisions regarding the choice between new, unfamiliar foods and familiar ones [25]. Therefore, neophobic consumers should not be overlooked during the new product development process, as they can sometimes constitute a significant part of the target market [26].

It is known that neophobic children consume fewer vegetables, fruits, and milk and dairy products than recommended [27,28,29]. There are fewer studies on the relationship between neophobia and food intake among adults than those on the children’s population, but a negative correlation between food neophobia (FNS score) and vegetable consumption was also found in adults [30,31]. It was also confirmed that higher food neophobia was associated with lower dietary quality, and neophobic individuals consumed a lower variety of food [30]. No association was found between the food neophobia and milk products (“Milk and Dairy Products” and “Cheese” dietary patterns) [32]. Moreover, food neophobes rarely consume functional and convenience foods [32,33], i.e., food that appears on the market as previously unknown to the consumer and may cause fear and discomfort when deciding to purchase it. Plant-based dairy substitutes, which can be a good complement to the diet and contribute to its variety, also represent foods that are still little known and therefore difficult to accept for neophobes, but also for other people [34,35,36]. Thus, food neophobia may not only make it difficult to purchase a variety of foods but also make it challenging to adhere to dietary recommendations, thereby increasing susceptibility to nutritional deficiencies and non-communicable chronic diseases.

The impact of food neophobia on food choices and eating behaviors can be reduced by exposure to a greater variety of foods, including new food products [37]. Therefore, when seeking methods to mitigate food-related concerns, the indirect presentation of food in various information sources should be considered. To date, only a few studies have investigated the relationship between food neophobia and interest in nutritional information, including that provided on food product labels [32,38,39]. The available results indicated that people with food neophobia searched for information on labels to determine whether a food product contained ingredients they usually avoided [39].

Given that little is known about the relationship between food neophobia and dairy consumption, or between food neophobia and information seeking about dairy products, this justifies the study. Furthermore, there is a need to focus on neophobia related to dairy products themselves, which may be strongly associated with dairy consumption and information seeking, and thus explain consumption. In this context, understanding the relationship between dairy consumption, attitudes toward new dairy products, and searching for information is crucial. The results will provide a better understanding of the determinants of individual dairy consumption, as well as the factors contributing to success in the dairy market, particularly concerning new dairy products. In turn, using available information about dairy products can positively influence purchasing decisions and encourage consumption, including new products entering the market. We hypothesize that: H1—Greater openness to new dairy products correlates with higher dairy consumption; H2—Greater concerns about new dairy products correlate with lower dairy consumption; H3—Searching for information correlates with attitudes towards new dairy products. Thus, the aim of the study was to assess the relationship between dairy product consumption, searching for information about dairy products, and attitudes toward new dairy products among adult Poles.

## 2. Materials and Methods

### 2.1. Study Design and Sample Collection

A cross-sectional survey was conducted in December 2024 and January 2025. The survey invitation explained the study’s aim and ensured data confidentiality and anonymity. Additionally, the invitation specified that participants must be 18 years of age or older to participate in the study. Participants were recruited from an online access panel designed to ensure a representative sample of the population. The database is administered by a research firm operating in accordance with the International Code of Market, Opinion, and Society Research (ESOMAR). The required sample size was estimated based on a population of approximately 38 million adults in Poland, a 95% confidence level, and a 4% margin of error, yielding a minimum sample size of 600. Before filling out the questionnaire, the respondent agreed to participate in the study by selecting one of two options: “yes” or “no”. Only those who marked “yes” answered the questionnaire. The entire study sample included 1006 Polish adults from all regions of the country. The sample’s selection criteria considered the Polish population’s representativeness in terms of the province, and then the sampling was quota-based according to gender, education, and place of residence.

### 2.2. Questionnaire

The frequency of consumption of milk and yogurts, cottage cheese, and cheese was rated using a 6-point frequency scale from less than once a month or never (1), 1–3 times a month (2), once a week (3), a few times a week (4), once a day (5) to a few times a day (6) [40]. Based on the frequency scale, the daily frequency was calculated. The values range from 0—less than once a month or never (1), 0.06—1–3 times a month (2), 0.14—once a week (3), 0.5—few times a week (4), 1—once a day (5) to 2—a few times a day (6) [41]. The result of summing the daily frequency of consumption of the three groups of dairy products was the daily frequency of dairy consumption. The scores ranged from 0 to 6, with a median of 1.14 and an interquartile range of 0.94. The higher the score, the more frequently dairy products are consumed during the day. Based on the median, two groups were identified: individuals who rarely consumed dairy products (below the median) and those who frequently consumed dairy products (at or above the median).

Nine statements from the Food Neophobia Scale—FNS [42] were used to assess attitudes towards new dairy products (Dairy Neophobia Scale—DNS): (1) I am constantly trying new and different dairy products; (2) I trust new dairy products; (3) When I’m unsure about the ingredients in dairy products, I avoid them; (4) I enjoy trying dairy products from various countries; (5) A dairy-free diet seems weird to me; (6) During parties, I enjoy trying new dishes that contain dairy products; (7) I am afraid to eat dairy products that I have not tried before; (8) I am very demanding about the new dairy products I eat; (9) I eat almost everything. The statement regarding the use of ethnic restaurants was omitted from the DNS, assuming that differences in the use of ethnic restaurants in the study group may significantly affect the results of neophobia towards new dairy products. The original FNS statements included the category “novel food” instead of “new dairy products.” Respondents expressed their opinion about each statement on a 5-point scale: 1 = strongly disagree; 2 = rather disagree; 3 = neither disagree nor agree; 4 = rather agree; 5 = strongly agree.

Respondents were also asked about the frequency of consulting various sources of information about dairy products, such as: family and friends; information on the packaging and/or product label; the Internet (e.g., portals devoted to nutritional issues); doctors, dietitians and nutrition specialists; culinary books and/or nutritional guides; culinary programs (e.g., television, radio); food retailers; social media; store websites; advertising (e.g., television, press, billboards); store leaflets; press articles; milk producers’ websites; and influencers. The frequency of consulting the above-mentioned information sources was measured on a 5-point scale, where 1 = never, 2 = rarely, 3 = neither rarely nor often, 4 = often, and 5 = very often.

### 2.3. Statistical Analysis

Nominal variables are presented as a percentage of the sample (%), and variables from ordinal scales are presented as medians with interquartile range (IQR) or means with standard deviation (SD). The Kolmogorov–Smirnov test was used to test the distribution of ordinal variables. The variables were not normally distributed, and a nonparametric test (Kruskal–Wallis test with Bonferroni correction) was used to compare mean values. For nominal variables, differences between groups were verified using the chi-square test. A significance level of *p* < 0.05 was considered significant for both tests.

An Exploratory Factor Analysis (EFA) was used to examine the nature of DNS. Based on eigenvalues (eigenvalues higher than 1), a 2-factor solution was suggested (Table 1). The factors obtained explained 52.0% of the total variation. Qualification of particular factors was based on a minimum factor loading value of 0.6. Factor selection was confirmed using the Kaiser-Meyer-Olkin (KMO) measure of sampling adequacy (KMO = 0.804) and Bartlett’s test of sphericity (*p* < 0.001). Cronbach’s alpha confirmed the reliability of DNS (0.716), and both identified factors, i.e., “Openness to new dairy products” (0.718) and “Concerns about new dairy products” (0.750). Within each factor, three categories were distinguished based on the tertile distribution of the regression coefficients: the first tertile (T1), the second tertile (T2), and the third tertile (T3). Participants in T3 represented the strongest adherence to the factor.

The identified factors were then used in cluster analysis to identify homogeneous groups based on attitudes toward new dairy products. The k-means clustering method was used. Cluster analysis identified three groups: “Moderate innovativeness and low concerns”; “Low innovativeness and medium concerns”; and “High innovativeness and high concerns.”

Statistical analysis was performed using IBM SPSS Statistics for Windows, version 29.0 (IBM Corp., Armonk, NY, USA).

## 3. Results

### 3.1. Characteristics of the Study Sample

The socio-demographic characteristics of the study group are presented in Table 1. The study group consisted of 1006 people aged 18–70 years (median 47; interquartile range 27).

### 3.2. The Daily Frequency of Dairy Consumption (DFD) in the Study Sample

The daily frequency of dairy consumption in the study group is presented in Table 2. The median was 1.14 (IQR = 0.94), with a range of 0 to 6. Milk and yogurt were consumed most frequently throughout the day. Cheese and cottage cheese consumption was at a similar level, and lower than the consumption of milk and yogurt.

Among the sociodemographic characteristics, only the age of the respondents differentiated the frequency of dairy product consumption (Table 3). Among people aged 60 years and over, there were more people who frequently (≥median) consumed dairy products (26.1%) than those who rarely (<median) consumed them (19.4%). In the 31–45 years age group, more people rarely consumed dairy products (33.9%) than frequently consumed them (28.6%). The remaining sociodemographic variables did not differentiate the frequency of dairy product consumption.

### 3.3. Dairy Product Consumption and Attitudes Towards New Dairy Products

The factor loading matrix for the identified factors representing attitudes towards new dairy products (DNS) is presented in Table 4. The identified factors are “Openness to new dairy products” and “Concerns about new dairy products”.

The highest percentage of people frequently consuming dairy products (median and above) was in the 3rd tertile of the “Openness to new dairy products” factor (38.9). The greater the openness to new dairy products, the fewer people rarely consumed dairy products (below the median). No differences in dairy product consumption were found after taking into account declared concerns about new dairy products (Factor 2) (Table 5).

Cluster analysis identified three groups: “Moderate innovativeness and low concerns” (41.2%), “Low innovativeness and medium concerns” (30.4%), and “High innovativeness and high concerns” (28.4%). Cluster names reflect the results for both factors extracted from DNS (Table 6).

The sociodemographic characteristics of the cluster, which comprise individuals with similar attitudes toward new dairy products, are presented in Table 7. The clusters differed in terms of gender, age, and education. The “Moderate innovativeness and low concerns” cluster consisted of more men, individuals aged 31–60, and those with vocational education. The “Low innovativeness and medium concerns” cluster included more women, individuals over 60 years old, and respondents with secondary and higher education. The “High innovativeness and high concerns” cluster consisted of more women, individuals aged 18–45, and respondents with primary education.

In the “Moderate innovativeness and low concerns” cluster, there was a similar number of people who consumed dairy products rarely (41.4%) and frequently (40.9%). In the “Low innovativeness and medium concerns” cluster, there were more people who rarely consumed dairy products (34.6%), while in the “High innovativeness and high concerns” cluster, there were more people who frequently consumed dairy products (32.1%) (Table 8).

### 3.4. Using Information About Dairy Products

The most common sources of information about dairy products consulted by respondents were family and friends, as well as information found on the packaging and/or product label, followed by the internet, doctors, dietitians and nutritionists, and cookbooks and/or nutritional guides (Table 9). People representing the “Innovative with high concerns” cluster used all sources of information more frequently than other clusters. The use of such information sources as family and friends, product packaging and/or label, Internet, doctors, dietitians and nutritionists, cookbooks and/or nutritional guides, cooking programs, sellers, shop websites, press articles, and milk producers’ websites did not differ in the “Moderate innovativeness and low concerns” and the „Low innovativeness and medium concerns” clusters. The use of social media, advertising, and influencers was most frequent in the „High innovativeness and high concerns” cluster and least frequent in the “Moderate innovativeness and low concerns” cluster (Table 9).

## 4. Discussion

The study’s results show that openness to new dairy products is associated with higher dairy consumption, regardless of concerns about these products, supporting hypothesis 1, while hypothesis 2 is not supported. In turn, openness to new dairy products, along with great concerns about them, was associated with more frequent use of specific information sources about dairy products. Thus, H3, indicating a relationship between attitudes towards new dairy products and information seeking, was confirmed. Furthermore, the significance of some sociodemographic characteristics in differentiating the variables was also noted.

Although many studies indicate the importance of sociodemographic factors in differentiating the consumption of dairy products [6,9,10,12], in the study group, only age was a variable with this effect. The differences observed in dairy product consumption across age groups are confirmed by previous studies [9,43,44]. Some studies show a decrease in dairy product consumption with age [9,44]. Although total dairy consumption decreases throughout life, the study by Cifelli et al. [44] showed that milk consumption itself increased slightly. Similarly, in the Chinese population, middle-aged adults consumed fewer dairy products than other age groups [43], but those over 60 years of age consumed higher amounts of milk [45]. However, the older consumers were characterized by a lower frequency of milk consumption compared to other age groups [46]. In our study, only the frequency of consumption was assessed, and it was found that among people over 60 years of age, there were more individuals who frequently consumed dairy products. The results of studies showing higher consumption in the elderly group can be explained by the fact that older people are more interested in maintaining health and preventing chronic diseases, and at the same time, perceive milk and dairy products as beneficial for health [47]. The fact that more people in the under-45 age group consumed dairy products less frequently may result from the questioning of the cultural position of milk and other dairy products for various reasons, including health risks [48] or climate change caused by intensive farming [49]. Inconsistent results regarding milk and dairy product consumption across different age groups and ethnic groups necessitate systematic monitoring of changes and their underlying causes [44].

The lack of differences in dairy product consumption across the study group based on other sociodemographic characteristics is surprising. As mentioned earlier, many individual characteristics are associated with dairy product consumption, including gender [6,50,51]. Although studies have been conducted that do not confirm this relationship for certain product groups [7], most find differences based on gender. Thus, higher milk consumption [8,52], as well as lactose-free milk consumption, was observed among women. Studies suggest that women’s higher knowledge about the health benefits of dairy products could lead to higher dairy consumption [9,53]. There are also studies available that show higher dairy product consumption [10] and higher consumption of cheese and milk-based desserts among men [52]. The lack of differences in dairy product consumption in this study group may be caused by the fact that the analyses did not account for individual dairy products.

Openness to new dairy products, as assessed using the Dairy Neophobia Scale (DNS), is indicative of consumer innovativeness. One approach to innovation identifies three categories of innovation: domain-specific innovativeness (innovativeness related to a product category), innate innovativeness (a personality trait that triggers new product trials), also referred to as global innovativeness [54], and innovative behavior [55]. Since the study only included milk and dairy products, domain-specific innovativeness refers to dairy food. DNS primarily refers to innovative behaviors, which are believed to be influenced by, among other things, the willingness to take action and the search for novelty [56]. In this study, trying new and different dairy products, as well as those from various countries, and eating almost everything as elements of the isolated factor (Openness to new dairy products) represent primarily innovative behaviors. However, trusting new dairy products is a component of innate innovativeness. The study revealed that openness to new dairy products led to more frequent consumption of dairy products, which may also be associated with a greater variety [57]. In a cluster characterized by both openness to and high concerns about new dairy products, there were more people who consumed dairy products more frequently, while in the “Low innovativeness and medium concerns” cluster, there were more people who rarely consumed dairy products. The lack of differences in the frequency of consumption of dairy products in view of the concerns about new dairy products may result from the fact that most decisions related to the purchase of dairy products are of a routine nature, and their consumption is habitual [57,58]. The association between high concerns about new dairy products and frequent dairy consumption seems illogical. However, high levels of concern may be shared by people who are attached to familiar dairy products and consume them frequently [58]. The results of previous studies show that consumers attach importance to ingredients they are familiar with and confident about their impact on health [59,60], as well as to the nutritional composition [61]. Moreover, concerns may arise from a lack of product knowledge [62], as well as from high expectations towards dairy products without being convinced that they will be met [63]. Product-related factors that concern consumers could influence dairy consumption [36,64], as could consumer innovativeness. This confirms that purchasing decisions regarding new dairy products are increasingly influenced by psychological factors, making them more personal and subjective [65,66]. The link between innovative behavior and frequent dairy consumption should be taken into consideration by dairy producers. Even if consumers do not exhibit a personality trait known as innate innovativeness, they could be encouraged to demonstrate innovative behavior to increase product sales.

The identification of clusters based on openness to and concerns about new dairy products, followed by profiling them based on dairy product consumption, provides an insight into the importance of socio-demographic characteristics, such as gender, age, and education, and psychosocial characteristics in determining dairy product consumption. The results suggest that women’s consumption of dairy products may be driven more by their openness to new products than by concerns about such products. Greater health awareness among women, particularly regarding the health benefits of dairy products [53], may explain the reduced influence of fears regarding dairy product consumption. Moreover, the low innovativeness of older people contributed to less frequent consumption of dairy products. More people with primary education were among the “High innovativeness and great concerns” cluster than in the other clusters. In comparison, more people with higher education were among the “Low innovativeness and medium concerns” cluster, which may be surprising because a positive relationship between innovation and education could be expected [67,68]. The results obtained in the study are not always consistent with the results of previous studies, which may suggest that the relationship between sociodemographic characteristics, openness to new products, and concerns about them differs depending on whether the latter two variables are considered separately or jointly (for example, as clusters). Therefore, searching for relationships between single variables when their effect is complex may have limited application in explaining the observed phenomena.

In the study sample, information about dairy products was sought from various sources, including traditional sources such as family and friends [61,69], sources provided by the market, i.e., cookbooks and/or nutritional guides [70] and the packaging and/or product label [71,72], sources represented by specialists, i.e., doctors, dietitians and nutritionists [73], but also internet sources [74,75,76,77]. Such a wide range of information sources provides great communication opportunities for people involved in the development of the dairy products market. However, some of them, i.e., advertising (e.g., television, press, billboards), shop leaflets, press articles, milk producers’ websites, and especially influencers, were indicated relatively less frequently in the study group. These sources primarily represent sources used by producers to communicate with consumers, which may hinder this communication and contribute to the generation of misunderstandings and concerns. A study by Paleologo et al. [78] confirmed gaps in perceptions of milk quality between groups present in the dairy market: farmers focused on economic issues, consumers on animal welfare and health, and processors on lactose content, which may additionally generate misunderstandings during communication. It can lead to a mismatch between consumer expectations and industry practices [79], contributing to consumer concerns about products. Thus, improving communication and understanding between these parties can lead to more effective strategies for resolving consumer concerns [78]. In this context, interactivity could help companies and consumers learn from each other about the practical use of products [80]. However, in the study group, the use of social media to search for information about dairy products was less important than direct contact with others (family, friends, dieticians, nutritionists, etc.), as well as with the product packaging and/or label and the Internet (e.g., portals devoted to nutritional issues). In particular, consulting social media, advertising, and influencers was least common in the “Moderate innovativeness and low concerns” cluster. Therefore, the obtained results do not confirm that social media is a powerful tool in consumer empowerment, in relation to dairy products [75,77]. This may be because dairy products do not generate enough interest to justify the publication of content on blogs and social media, which, in turn, help answer customers’ questions and make consumption decisions [81,82]. People who represented the „High innovativeness and high concerns” cluster used all sources of information about dairy more often than other clusters. This means that seeking information about dairy products may be linked to both innovativeness and concerns. The latter can be interpreted in various ways. Concerns may prompt a search for information to clarify them, but excess and diversity of available information may also raise concerns [83,84].

The results of this study may have practical implications for dairy producers and marketing experts. Differences in consumer behavior stem in part from their openness to new products, which is particularly important in the case of dairy products. In the dairy market, purchasing decisions are typically routine, and purchasing behavior is habitual. Therefore, encouraging innovative behavior can lead to greater dairy product consumption. Therefore, there is a need to develop strategies that increase consumer openness to new offerings. Awareness of differences in both food consumption and attitudes toward new dairy products, depending on sociodemographic characteristics, should help improve the effectiveness of such initiatives. This can be helpful in developing marketing strategies targeted at specific consumer groups. The results can also be used in educational and promotional campaigns encouraging the consumption of dairy products as part of a balanced diet. Future research should include other population groups representing different cultural contexts to expand our understanding of global differences in view of the importance of attitudes toward new dairy products in determining dairy consumption. The results describing the relationship between concerns about new products, the frequency of their consumption and information about dairy products suggest potentially more complex relationships, including, among others, the mediating effect of information, which should encourage researchers to continue this line of research due to their practical applications. Furthermore, continued research should examine the importance of information from various sources for consumers, considering both its use and its content. The findings could be valuable to both the scientific community and the dairy industry.

### Strengths and Limitations

The study’s strength lies in its large, representative sample of Polish adults. However, the sample was drawn from an online panel, which, although stratified to reflect national demographics, may underrepresent individuals without internet access or those less inclined to participate in surveys. This may introduce selection bias, favouring more engaged consumers. Moreover, findings are specific to the Polish cultural background and should be used with caution in relation to other populations. The study’s limitations include the large proportion of self-reported data [85] and its cross-sectional design, which did not allow for assessing the causality of relationships between the variables. It only allows for the consideration of correlations between variables, which significantly limits the possibilities of interpreting the results.

The study of the relationship between various factors and the consumption of dairy products measured by the frequency of consumption is also limited due to the possibility of overestimating the consumption of some products when measuring them in this manner [86]. Therefore, the study’s results refer solely to consumption frequency patterns and should be interpreted accordingly. They do not account for quantitative intake and do not address nutritional value. The scale for measuring attitudes toward new dairy foods (DNS) was constructed based on the FNS. The DNS was not validated prior to the study. Even though Cronbach’s alpha confirms its reliability, the exploratory nature of the DNS is a limitation of the study. Another limitation of the study is that it primarily considers innovative behaviors as components of consumer openness to new dairy products, with only a small share of innate innovativeness, which may exhibit high variability depending on the individual and culture [87]. Although the findings should not be generalized to populations with a different cultural background, our study provides an interesting insight into the relationships between dairy product consumption, use of information about dairy products, and attitudes toward new dairy products. This may be particularly important for dairy producers introducing new dairy products to the market, as well as for educators and individuals involved in various nutritional interventions aimed at improving dietary habits.

## 5. Conclusions

The study results indicate that consumers’ attitudes towards new dairy products and their engagement with available information about dairy products are related to their consumption of dairy products. Openness to new dairy products was associated with higher dairy product consumption, regardless of the level of concern about these products. Therefore, there is a need to take action to support consumers’ willingness to try new things, even if they do not demonstrate innate innovativeness. However, it should be noted that, in addition to openness to new dairy products, the level of concern about new dairy products was also related to the use of various sources of information. Openness to and greater concern about new dairy products encouraged more frequent use of all sources of information. Simultaneously, the use of various information sources was associated with higher consumption of dairy products. There is, therefore, a need to develop more effective methods of reaching consumers with information about new dairy products, especially those with less favorable attitudes characterized by lower openness and less concern about new dairy products, so that their attitudes can support the development of the dairy food market.

## Figures and Tables

**Table 1:** Socio-demographic characteristics of the study sample.

Socio-Demographic Characteristics	Total Sample % (*n*) *
Total sample	100.0 (1006)
Gender	
Female	52.8 (531)
Male	47.2 (475)
Age (in years)	
18–30	21.3 (214)
31–45	31.0 (312)
46–60	24.7 (248)
More than 60	23.1 (232)
Education	
Primary	18.8 (189)
Vocational	22.9 (230)
Secondary	29.2 (294)
Higher	29.1 (293)
Place of residence	
A village	37.9 (381)
A town with fewer than 20,000 inhabitants	12.9 (130)
A city with 20,000–100,000 inhabitants	20.5 (206)
A town with 100,001–500,000 inhabitants	15.2 (153)
A city with over 500,000 inhabitants	13.5 (136)
Self-reported financial situation	
Very good—there is enough for everything without much saving	5.7 (57)
Good—I live frugally, and I have enough means for everything	31.0 (312)
Average—I live very frugally to save for major purchases	53.2 (535)
Rather bad—I have to be very frugal daily	8.3 (83)
Bad—not enough even for basic needs	1.9 (19)

* *n*—number of participants.

**Table 2:** Statistics on the daily consumption of dairy products in the study group.

		Cottage Cheese		Cheese		Milk and Yogurts		Dairy Products—Total	
		Statistics	St. Error	Statistics	St. Error	Statistics	St. Error	Statistics	St. Error
Mean		0.33	0.011	0.38	0.012	0.63	0.017	1.33	0.030
95% confidence interval for the mean	Lower limit	0.31		0.35		0.60		1.27	
	Upper limit	0.35		0.40		0.66		1.39	
Median		0.14		0.15		0.50		1.14	
Variance		0.13		0.15		0.28		0.90	
Std. dev.		0.36		0.39		0.53		0.95	
Minimum		0.00		0.00		0.00		0.00	
Maximum		2.00		2.00		2.00		6.00	
Range		2.00		2.00		2.00		6.00	
Interquartile range		0.36		0.44		0.86		0.94	
Skewness		2.27	0.077	2.14	0.077	1.33	0.077	1.64	0.077
Kurtosis		6.88	0.154	6.23	0.154	1.30	0.154	4.15	0.154

**Table 3:** Daily frequency of dairy consumption according to participants’ age.

Sociodemographic Characteristics	Total Sample % (*n*) *	Daily Frequency of Dairy Consumption		*p*-Value **
		Low (<Median) % (*n*)	High (≥Median) % (*n*)	
Total sample	100.0 (1006)	45.1 (454)	54.9 (552)	
Age (years)				
18–30	21.3 (214)	22.5 (102)	20.3 (112)	0.050
31–45	31.0 (312)	33.9 (154)	28.6 (158)	
46–60	24.7 (248)	24.2 (110)	25.0 (138)	
More than 60	23.1 (232)	19.4 (88)	26.1 (144)	

* *n*—number of participants; ** *p*-value—level of significance (Chi-square test).

**Table 4:** Factor loading matrix for factors identified based on EFA.

Statements from the Dairy Neophobia Scale (DNS)	Factors	
	Factor 1 (Openness to New Dairy Products)	Factor 2 (Concerns About New Dairy Products)
I am constantly trying new and different dairy products.	0.751 *	0.182
I trust new dairy products.	0.730 *	0.067
When I’m unsure about the ingredients in dairy products, I avoid them.	0.007	0.779 *
I enjoy trying dairy products from various countries.	0.754 *	−0.001
A dairy-free diet seems weird to me.	0.454	0.269
During parties, I enjoy trying new dishes that contain dairy products.	0.781 *	0.026
I am afraid to eat dairy products that I have not tried before.	−0.077	0.738 *
I am very demanding about the new dairy products I eat.	0.218	0.708 *
I eat almost everything.	0.608 *	−0.125
% of variance explained	33.4	18.6

* Correlation coefficient > 0.6.

**Table 5:** Consumption of dairy products in the study sample, taking into account attitudes towards new dairy products (the identified factors).

The Identified Factors		Daily Frequency of Dairy Consumption		Total
		Low (<Median) % (*n*)	High (≥Median) % (*n*)	
Total		100.0 (454)	100.0 (552)	100.0 (1006)
Openness to new dairy products (Factor 1) (*p* < 0.001) *	1st tertile	43.4 (197)	29.9 (165)	36.0 (362)
	2nd tertile	30.2 (137)	31.2 (172)	30.7 (309)
	3rd tertile	26.4 (120)	38.9 (215)	33.3 (335)
Concerns about new dairy products (Factor 2) (*p* = 0.563) *	1st tertile	34.6 (157)	32.2 (178)	33.3 (335)
	2nd tertile	31.7 (144)	34.8 (192)	33.4 (336)
	3rd tertile	33.7 (153)	33.0 (182)	33.3 (335)

* *p*-value—level of significance (Chi-square test).

**Table 6:** Cluster characteristics according to regression factor scores.

Identified Clusters	Regression Factor Scores (Mean, SD)	
	Factor 1 Openness to New Dairy Products (Innovativeness)	Factor 2 Concerns About New Dairy Products
Moderate innovativeness and low concerns	0.16 (0.740)	−0.88 (0.627)
Low innovativeness and medium concerns	−0.99 (0.709)	0.45 (0.681)
High innovativeness and high concerns	0.83 (0.631)	0.79 (0.701)
*p*-value	<0.001	<0.001

**Table 7:** Cluster characteristics according to sociodemographic characteristics of the study sample.

Sociodemographic Characteristics	Total Sample % (*n*) *	Clusters			*p*-Value **
		Moderate Innovativeness and Low Concerns % (*n*)	Low Innovativeness and Medium Concerns % (*n*)	High Innovativeness and High Concerns % (*n*)	
Total sample	100.0 (1006)	41.2 (414)	30.4 (306)	28.4 (286)	
Gender					
Female	52.8 (531)	45.2 (187)	62.1 (190)	53.8 (154)	<0.001
Male	47.2 (475)	54.8 (227)	37.9 (116)	46.2 (132)	
Age (years)					
18–30	21.3 (214)	21.3 (88)	18.6 (57)	24.1 (69)	<0.001
31–45	31.0 (312)	33.0 (137)	23.5 (72)	36.0 (103)	
46–60	24.7 (248)	26.1 (108)	24.8 (76)	22.4 (64)	
More than 60	23.1 (232)	19.6 (81)	33.1 (101)	17.5 (50)	
Education					
Primary	18.8 (189)	19.8 (82)	14.1 (43)	22.4 (64)	<0.001
Vocational	22.9 (230)	25.8 (107)	17.3 (53)	24.5 (70)	
Secondary	29.2 (294)	27.8 (115)	36.6 (112)	23.4 (67)	
Higher	29.1 (293)	26.6 (110)	32.0 (98)	29.7 (85)	

* *n*—number of participants; ** *p*-value—level of significance (Chi-square test).

**Table 8:** Consumption of dairy products, taking into account the identified clusters (*p* < 0.05) *.

Clusters	Daily Frequency of Dairy Consumption		Total % (*n*)
	<Median % (*n*)	≥Median % (*n*)	
Moderate innovativeness and low concerns	41.4 (188)	40.9 (226)	41.2 (414)
Low innovativeness and medium concerns	34.6 (157)	27.0 (149)	30.4 (306)
High innovative and high concerns	24.0 (109)	32.1 (177)	28.4 (286)
Total	100.0 (454)	100.0 (552)	100.0 (1006)

* Chi-square test.

**Table 9:** Using various sources of information on dairy products in the study sample, taking into account the affiliation to clusters.

Frequency of Use of Information Sources	Total	Clusters		
		Moderate Innovativeness and Low Concerns	Lowly Innovativeness and Medium Concerns	High Innovativeness and High Concerns
Family and friends		455.7 a *	465.8 a	613.0 b
	3; 1 **	3; 1	3; 1	4; 1
Information on the product packaging and/or label		461.0 a	470.5 a	600.3 b
	4; 1	3; 1	3; 2	4; 2
Internet (e.g., portals devoted to nutritional issues)		468.7 a	426.1 a	636.7 b
	3; 2	3; 2	3; 2	4; 1
Doctors, dietitians and nutritionists		457.5 a	447.1 a	630.4 b
	3; 2	3; 2	3; 2	4; 1
Cookbooks and/or nutritional guides		458.2 a	439.7 a	637.4 b
	3; 2	3; 2	3; 2	4; 1
Cooking programs (e.g., television, radio)		468.7 a	427.7 a	634.9 b
	3; 2	3; 2	3; 1	4; 1
Sellers		458.5 a	425.2 a	652.4 b
	3; 2	3; 1	2; 1	3; 1
Social media		480.1 a	402.0 b	645.9 c
	3; 2	3; 1	2; 2	3; 1
Shop websites		451.5 a	421.3 a	664.6 b
	3; 1	2; 1	2 a; 2	3 a; 1
Advertising (e.g., television, press, billboards)		480.1 a	415.3 b	631.8 c
	3; 1	3; 1	2; 2	3; 2
Shop leaflets		460.9 a	426.4 a	647.6 b
	3; 1	2; 1	2; 2	3; 2
Press articles		457.9 a	415.6 a	663.5 b
	3; 1	2; 1	2; 2	3; 2
Milk producers’ websites		456.1 a	419.0 a	662.5 b
	3; 2	2; 1	2; 2	3; 1
Influencers		479.4 a	402.4 b	646.7 c
	2; 2	2; 2	1; 1	3; 2

* mean rank from a 5-point scale, where 1—never, 2—rarely, 3—neither rarely nor often, 4—often, 5—very often; a,b,c values marked with different letters differ statistically (*p* < 0.05; Kruskal–Wallis test with Bonferroni correction) ** median; IQR.

## Data Availability

The data presented in this study are available on request from the corresponding author because the data have not yet been made available in publicly available databases.
